# Modified Maimendong decoction in the treatment of patients with idiopathic pulmonary fibrosis: Study protocol for a randomized controlled trial

**DOI:** 10.1097/MD.0000000000023460

**Published:** 2020-12-04

**Authors:** Wenfan Gan, Qun Huang, Guojin Xiao, Ying Luo, Jundong Wang, Chuantao Zhang, Yuancheng Liang, Niao Huang, Tingting Liao

**Affiliations:** a Department of Respiratory Medicine; b Department of Ophthalmology, Hospital of Chengdu University of Traditional Chinese Medicine; c Hospital of Chengdu University of Traditional Chinese Medicine; d Sichuan Civil Administration Rehabilitation Hospital; e Chengdu University of Traditional Chinese Medicine; f Department of Endocrinology, Hospital of Chengdu University of Traditional Chinese Medicine, Chengdu, China.

**Keywords:** idiopathic pulmonary fibrosis, modified Maimendong decoction, randomized controlled trial, traditional Chinese medicine

## Abstract

**Introduction:**

With dissatisfaction of western medicine, traditional Chinese medicine becomes alternative treatment for idiopathic pulmonary fibrosis patients. The common syndrome of idiopathic pulmonary fibrosis (IPF) is qi and yin deficiency syndrome. The prescription, Modified Maimendong Decoction (MMD), is usually used for IPF patients with qi and yin deficiency syndrome. However, there is no convinced evidence for the efficacy and safety of MMD to treat IPF.

**Methods:**

A double-blind, placebo-controlled, randomized clinical trial was put forward by us. After a 1-day run-in period, 60 eligible patients will be included in the study. These subjects will be allocated to the experiment group or control group in a 1:1 ratio. Patients in the experiment group will take MMD plus Pirfenidone capsule. At the same time, patients in the control group will receive a matched placebo plus Pirfenidone capsule. All subjects will receive 24 weeks of treatment and follow-up period. The primary outcomes are the mean change from the baseline in forced vital capacity and times of acute exacerbations at week 4, 12, 24. Secondary outcomes are the mean change from baseline in the St. George's respiratory questionnaire total score, forced expiratory volume in 1 second percentage/forced vital capacity, diffusing capacity of Carbon monoxide, brain natriuretic peptide, and curative effect of traditional Chinese medicine syndrome at week 4, 12, and 24. Any side effects of the treatment will be recorded.

**Discussion:**

The results of this trial will provide the evidence for the effect of MMD in patients with idiopathic pulmonary fibrosis.

## 1 Introduction

Idiopathic pulmonary fibrosis (IPF) is a disease characterized by diffuse alveolitis and alveolar structural disorders that eventually lead to pulmonary fibrosis. The pathological and radiological manifestations of idiopathic pulmonary fibrosis are usual interstitial pneumonia. The main clinical manifestations are unexplained chronic dyspnea, cough, bursting sound at the base of both lungs and/or clubbing fingers. The prognosis of idiopathic pulmonary fibrosis is poor, and the median survival time after diagnosis is only 3 to 5 years.^[1]^ In the course of the disease, it can lead to many complications such as emphysema, pulmonary hypertension, lung cancer, venous thromboembolism.^[2]^ In the progression of the disease, it may eventually develop into respiratory failure and die. Epidemiological investigation found that the incidence of IPF is 2 to 29/100,000.^[3,4]^ The results of a systematic review showed that the incidence of IPF was 2.8 to 9.3/100,000, and 3 to 9/100,000 in North America and Europe, which was higher than that in South America and Asia (<4/100,000).^[5]^ With the development of medical technology, the diagnosis rate and incidence of IPF are increasing year by year, which not only brings serious health problems to patients, but also brings heavy economic burden to the society.^[6]^ At present, there is no specific drug for the treatment of IPF in western medicine. In 2016, Chinese experts agreed on the diagnosis and treatment of idiopathic pulmonary fibrosis^[7]^ to recommend antacid drugs such as Pyrifenidone and Nidanib to treatment, but it is difficult to control the occurrence and development of IPF. Therefore, to further find a solution for the prevention and treatment of IPF, reduce the medical cost and reduce the social burden has become an important task for the prevention and treatment of the disease.

Traditional Chinese medicine (TCM) has the characteristics of multi-targets in the treatment of IPF and plays an important role in the adjuvant treatment of IPF. IPF belongs to the disease name of western medicine, and there is no corresponding disease name in the ancient books of TCM. At present, the disease can be classified as “lung arthralgia (a disease of the lung due to external pathogen blocking lung qi, marked by dyspnea and vomiting, chest and back pain)” and “lung withered (a disease of the lung due to chronic cough, marked by atrophy of the lung with shortness of breath and expectoration)” in TCM according to the clinical symptoms, etiology and pathogenesis of IPF. The pathogenesis of “pulmonary arthralgia” is consistent with IPF in western medicine. In the process of IPF, the capillaries in the lung proliferate and dilate, the wall thickens, a large number of fibrous connective tissue proliferates and the blood vessels contract, and the number of capillaries decreases or even atresia.^[8]^

Using Chinese medicine in conjunction with Western medicine showed greater efficacy than using the Western medicine alone in delaying the decline of pulmonary function and quality of life with good safety. A large number of experiments have confirmed that TCM prescription is effective in the auxiliary treatment of IPF. Ren Luyao et al^[9]^ used Yiqi Huoxue Guben recipe combined with acetylcysteine granule to treat idiopathic pulmonary fibrosis. The results showed that Yiqi Huoxue Guben recipe could delay the decline of lung function, improve respiratory symptoms, increase activity endurance and improve the quality of life. It is effective in the clinical treatment of idiopathic pulmonary fibrosis. Yan Jingjing et al,^[10]^ who treated idiopathic pulmonary fibrosis with Qingfei Huaxian decoction combined with western medicine, concluded that Qingfei Huaxian decoction in patients with IPF can effectively reduce clinical symptoms, improve pulmonary function, reduce the level of pulmonary fibrosis indexes (serum laminin, hyaluronic acid), and has high safety. Xue Honghao et al^[11]^ did a Clinical study which has shown that chemical fiber decoction can reduce Borg dyspnea score and St. George's respiratory questionnaire quality of life score in patients with idiopathic fibrosis, as well as serum TNF-α and TGF-β levels. It is concluded that chemical fiber decoction may play a therapeutic role in patients with IPF by inhibiting inflammatory reaction. Yuan Jie's Clinical trial results show that Xuefu Zhuyu decoction combined with N-acetylcysteine has a good effect on inhibiting the progression of idiopathic pulmonary fibrosis, can effectively alleviate inflammatory reaction, improve lung function, and has high safety.^[12]^ Duan Chenxia et al^[13]^ found that Peiyuan Quyu recipe has a significant effect on improving the clinical symptoms of IPF of phlegm and blood stasis, but also can improve the index of pulmonary function, reduce the acute aggravation of the disease, significantly improve the exercise endurance and quality of life, and the curative effect is reliable. Zhang Wei et al^[14]^ by using Qingjin Yifei decoction to treat rats with idiopathic pulmonary fibrosis of phlegm-heat accumulation in the lung, they found that Qingjin Yifei decoction could reduce the expression of VEGFmRNA and PDGF, thus reduce capillary permeability, dilate pulmonary arteries and veins, and improve vascular remodeling. Through clinical observation and animal experiments, Zhang Guicai et al^[15]^ found that Huqi Huoxue decoction can reduce the deposition of collagen in the lungs and improve the symptoms of patients with idiopathic pulmonary fibrosis by inhibiting the expression of TGF- β and its protein. MengLi^[16]^ found that Yangqing Kangxian recipe has a good protective and long-term effect on pulmonary fibrosis by anti-inflammation and reducing fibrosis.

Maimendong decoction comes from *the Synopsis of the Golden Chamber* (JinGui YaoLue) written by Zhang Zhongjing in the Han Dynasty. The prescription is composed of Radix Ophiopogonis japonica (Maimendong), Pinellia ternate (Banxia), Radix Ginseng (Renshen), Licorice (Gancao), Japonica rice (Jingmi), Jujube (Dazao). Chinese researchers have done a large number of experiments to confirm the efficacy of Maimendong decoction in the treatment of idiopathic pulmonary fibrosis. A rat experimental study found that Radix Ophiopogonis japonica can reduce the degree of pulmonary fibrosis in rats with IPF, probably by increasing the level of BMP-4.^[17]^ Liu Xiaoming et al^[18]^ showed that Pinellia ternata had the same effect as pirfenidone in improving Nrf2 antioxidant index and regulating antioxidant gene expression. Pinellia ternata can interfere with pulmonary fibrosis by interfering with the mechanism of oxidation-antioxidation. Some studies have shown that ginsenoside 1 (Rg1), the main active component of Panax ginseng, has a protective effect on bleomycin-induced pulmonary fibrosis in rats, and its mechanism may be related to down-regulation of transforming growth factor β-1 and upregulation of Cav-1.^[19]^ Some experiments have found that compound glycyrrhizin, an effective component of licorice, can reduce pulmonary fibrosis in rats with IPF, and its mechanism may be related to regulating the level of free radicals and reducing the oxidative damage of free radicals to lung tissue structure.^[20]^ These experiments provide a pharmacological basis for Maimendong decoction in the treatment of IPF. The use of Modified Maimendong Decoction (MMD) in TCM has accumulated a great deal of clinical experience in the adjuvant treatment of IPF. Bai Wenmei^[21]^ concluded that the treatment of IPF patients with Maimendong decoction combined with pirfenidone can significantly relieve clinical symptoms, improve lung function and quality of life, and effectively delay pulmonary fibrosis by a clinical trial. The clinical trial of Yu long^[22]^ showed that Jiawei Maimendong decoction combined with conventional western medicine can reduce the symptoms and improve lung function of patients with IPF. A literature review shows that^[23]^ Maimendong decoction is effective in the treatment of pulmonary fibrosis, but its mechanism and safety of clinical application need to be further explored. These clinical studies have confirmed that MMD can treat IPF, but the quality of the literature is generally low. There is still a lack of high-quality evidence-based medicine to confirm the clinical efficacy and safety of MMD in the treatment of IPF. Therefore, we designed this randomized controlled trial to evaluate the clinical efficacy and safety of MMD in adjuvant treatment of IPF.

## 2 Methods

### 2.1 Study design

**Figure 1:**
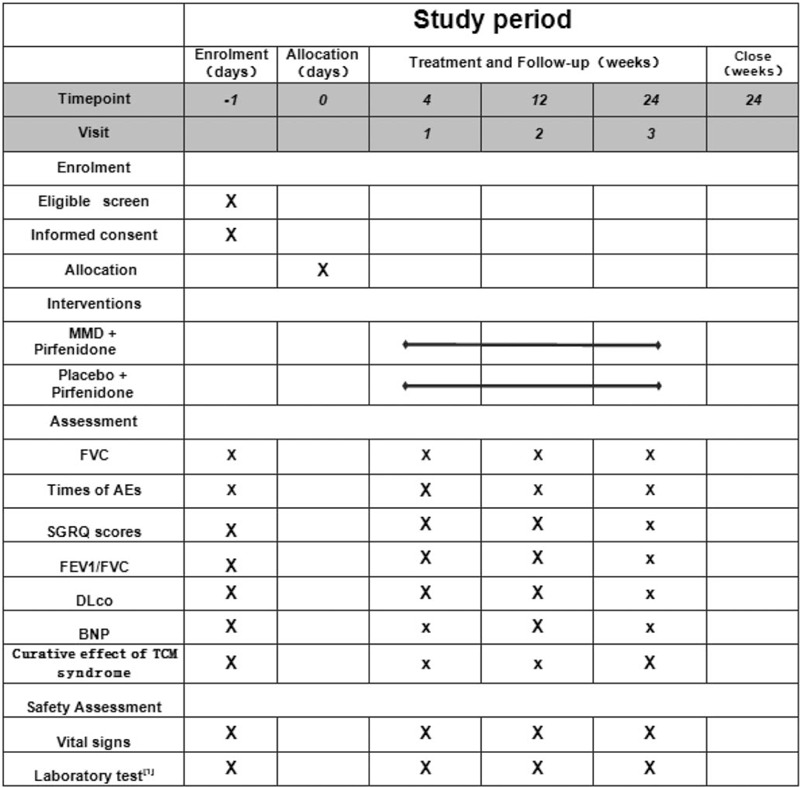
SPIRIT figure of enrollment, interventions, and assessments.^[1]^ Laboratory tests: blood, urine, feces, electrocardiogram, and kidney and liver function tests. BNP = brain natriuretic peptide, DL_CO_ = diffusing capacity of Carbon monoxide, FEV1 = forced expiratory volume in 1 second percentage, FVC = forced vital capacity, MMD = modified Maimendong decoction, SGRQ = St. George's respiratory questionnaire, SPIRIT = standard protocol items recommendations for interventional trials, TCM = traditional Chinese medicine.

**Figure 2:**
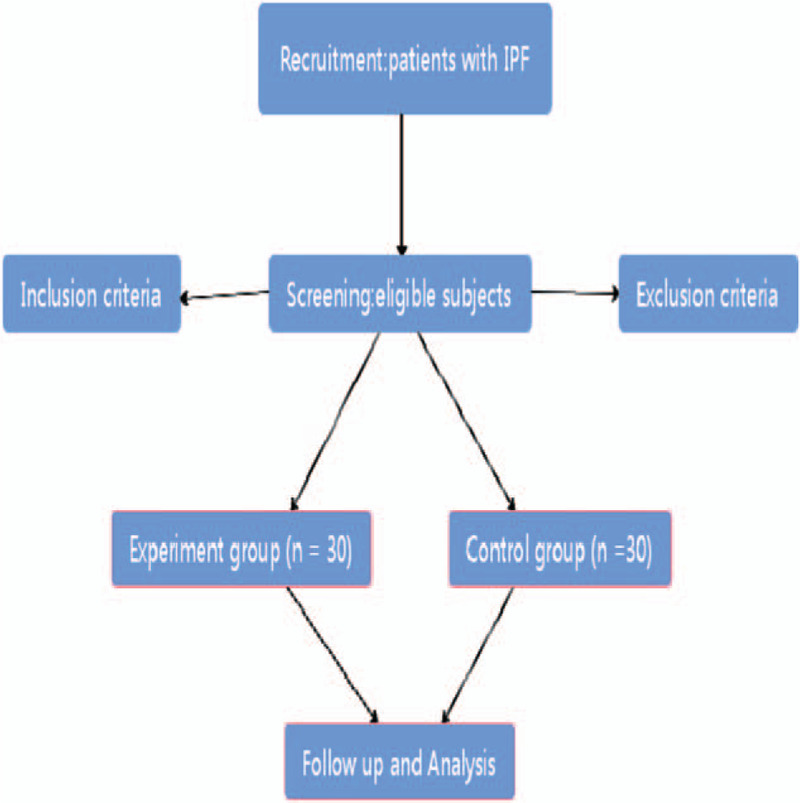
Flow chart of the study design.

An independent committee, including clinicians of respiratory department, statisticians and ethics experts, will be set up to perform the study design. A randomized, prospective, double-blind placebo-controlled clinical trial will be carried out to evaluate the efficacy and safety of MMD. This trial has been registered with the Chinese Clinical Trial Registry (no ChiCTR2000036021, registered on August 21th, 2020). This study will act in accordance with the Standard Protocol Items: Recommendations for Interventional Trials 2013 statement (see Fig. 1). Figure 2 shows a flowchart of the study.

### 2.2 Ethics approval and consent to participate

The study protocol will has been approved by the China Ethics Committee of Registering Clinical Trials in September 2020 and has been registered on the Chinese Clinical Trial Registry (http://www.chictr.org.cn/showprojen.aspx?proj=59112) with number ChiCTR2000036021 on August 21th, 2020. This trial will follow the principles of the Declaration of Helsinki and the International Ethical Guidelines for Biomedical Research Involving Human Subjects. Only when subjects provide informed consent, can they take part in this study.

### 2.3 Study population and recruitment

IPF patients will be recruited by respiratory physicians from respiratory clinics at the Hospital of Chengdu University of Traditional Chinese Medicine (Chengdu, China), a national clinical trial and research center of TCM. Subjects will be recruited via all kinds of method like advertisement and doctor referrals. All patients have to get the advice by a specialist in Department of Respiratory Medicine before receiving treatment. All the patients will sign the informed consent before enrollment. Recruitment will last for a year from September 2020 or until the total sample are completed.

### 2.4 Sample size calculation

Calculation of the sample size was based on the primary outcome variable, forced vital capacity (FVC). According to previous study,^[20]^ the mean of FVC in control group was 3.07 L and the standard deviation was 0.71 L after taking Pifenidone. With oral MMD plus Pifenidone, the mean in experiment group was 3.81 L, and the standard deviation was 0.85 L. We employed following formula to calculate the sample size in each group: n *= (μ*_*α*_* + μ*_*β*_*)*^*2*^ *×* *(1+1/k)* *×* *σ*^*2*^*/ (μ*_*2*_*-μ*_*1*_*)*^*2*^, where *σ*^2^ is overall variance, which was estimated as sample variance *s*^2^ just like the formula *s*^*2*^*= (s*_*1*_^*2*^* + ks*_*2*_^*2*^*) / (1 + k)*. The proportion of subjects between the experiment and control groups was set to 1:1 (k = 1). The study was designed to have a power of approximately 90% and 1-side level of significance of 0.05 (a = 0.05, β = 0.1). *μ*_2_, *μ*_1_, *s*_1_, and *s*_2_ are mean and standard deviations in the control and experiment groups respectively. Twenty-three patients are needed in each group based on the formula mentioned above. Considering a 10% of drop-out rate, approximately 26 patients should initially be recruited in each group. In the study, 30 subjects will be recruited in each group actually, with a total of 60 subjects.

### 2.5 Randomization and binding

A completely random design will be used to allocate patients who meet inclusion criteria and sign the informed consent. All the patients will be assigned to the 2 groups randomly in a 1:1 ratio. Patient enrollment, randomization and treatment allocation will be performed by 3 different researchers respectively. Random numbers (from 1 to 60) will be generated by a statistical expert of the Sichuan TCM Evidence-Based Medicine Center before recruitment, and then the numbers will be allocated to the 2 study groups. These numbers will be kept by an independent researcher in an opaque envelope with corresponding treatment allocation. Patients will be given MMD granules or placebo during the treatment period after grouped. Both MMD granules and placebo granules will be produced by Sichuan Green Pharmaceutical Technology Development Co, Ltd to ensure that they are identical in specifications such as appearance, shape, smell. All the patients and researchers (except for who conducting treatment allocation) will be all blind to group number and treatment plan. In case of an emergency, such as adverse events, the randomization number and treatment allocation will be exposed to researchers and supervisors as soon as possible. Therefore, the concealment and blinding of the assignment will be confirmed.

### 2.6 Diagnostic criteria

Subjects must meet the western medicine diagnostic criteria for IPF of ATS/ERS/JRS/ALAT in 2018 (Table 1)^[24]^ and the TCM syndrome diagnostic criteria of qi and yin deficiency syndrome (Table 2).^[25,26]^ The syndrome will be differentiated by 2 experienced and designated deputy physicians of TCM independently.

**Table 1:** Western medicine diagnostic criteria for IPF.

Diagnostic criteria for IPF
1. Exclusion of other known causes of ILD (eg, domestic and occupational environmental exposures, CTD, drug toxicity), and either #2 or #3;
2. The presence of the HRCT pattern of UIP;
3. Specific combinations of HRCT patterns and histopathology patterns in patients subjected to lung tissue sampling.

UIP = usual interstitial pneumonia

**Table 2:** The TCM syndrome diagnostic criteria for qi and yin deficiency syndrome.

Diagnostic Criteria for qi and yin deficiency syndrome
Primary symptoms:
(1) Dry cough with less phlegm;
(2) Breathless will be aggravated after moving.
Secondary symptoms:
(1) Low and weak cough voice;
(2) Fatigue and shortness of breath;
(3) Spontaneous sweating and fear of wind;
(4) Dry mouth and thirst;
(5) Night sweats.
Tongue and pulse: the tongue is tender and red with eroded fur (complete or partial peeling of the tongue coating), and the pulse is feeble and rapid or the pulse is small and weak.
More than 2 primary symptoms + 1 secondary symptom or 1 primary symptom + 2 secondary symptoms can be diagnosed as qi and yin deficiency syndrome.

TCM = traditional Chinese medicine.

### 2.7 Inclusion criteria and exclusion criteria

The inclusion criteria will be: subjects aged 18 to 75, male and female; Diagnosis according to guidelines of the Idiopathic Pulmonary Fibrosis by ATS/ERS/JRS/ALAT in 2018; in line with the diagnostic criteria of qi and yin deficiency syndrome of TCM; good ability to understand and write research-related materials, and voluntary compliance with all requirements of the study; volunteer to this study and sign an informed consent.

The inclusion criteria will be: patients with acute exacerbation of IPF, those with FVC% < 50% or those with obvious pulmonary infection should be treated with anti-infection therapy; patients with chronic pulmonary diseases (such as chronic obstructive pulmonary disease, interstitial lung disease, tuberculosis and bronchiectasis); patients with severe condition of mental disorder, or laboratory test suggesting severe systemic disease (such as the dysfunction of cardio-cerebrovascular, liver and kidney, endocrine, hematopoietic system or tumor); patients who had taken glucocorticoid, immunosuppressants or antacid within 1 month; unwilling or unable to change the current treatment plan; those who are known to be allergic to Chinese herb in the experimental drug; women who are lactating, pregnant or preparing for pregnancy; participated in other clinical studies in the past half a year.

### 2.8 Suspension and exit criteria

Patients will have the right to exit from this study. They will be treated with conventional treatment still once they withdraw the trial. Any reason of subjects exiting will be recorded in their case report file. The criteria of suspension and exit in the study will be as follows: there are serious adverse events or complications during the clinical trial; the without good compliance, subjects use drugs prohibited in this study; researchers argue that the trial may be harmful to the patient's body, mind, economy; the subjects, unwilling to the study, request to suspend the clinical trial.

### 2.9 Chinese herbal medicine

The prescription in experiment group is MMD, while MMD mimetic agent will be placebo in control group. All the Chinese medicine will be produced by the Sichuan Green Pharmaceutical Technology Development Co, Ltd (Sichuan, China). Chinese herbs of MMD are Radix Ophiopogonis japonici (Maimendong) 10 g, Pinellia ternate (Banxia) 15 g, Radix Ginseng (Renshen) 5 g, Polygonum cuspidatum (Huzhang) 10 g, Ligusticum (Chuanxiong) 10 g, Salviae Miltiorrhizae Radix Et Rhizoma (Danshen) 15 g and licorice (Gancao) 5 g. The herbs in the formula are manufactured to granules by the company. All the drugs will be consistent with the national standards of quality. The granules of each formula are divided into 3 parts equally, and then packaged into 3 small sachets. Placebos will be made of non-active ingredients. The placebo will be identical with the MMD granules in characteristics such as weight, appearance, smell and taste.

## 3 Intervention

### 3.1 Treatment plan

The subjects in both groups will be treated with routine western medicine, and the treatment plan will be based on the consensus of Chinese experts in the diagnosis and treatment of IPF^[7]^: Pirfenidone capsule (trade name: Esri specification: 100 mg ∗ 54 manufacturer: Beijing Contini Pharmaceutical Co., Ltd.). In the first week, 200 mg each time will be taken 3 times a day for a week. In the second week, 400 mg each time will be taken 3 times a day for 4 weeks. In the sixth week, 600 mg each time will be taken 3 times a day for long-term maintenance.

Experiment group: subjects in the experimental group will receive MMD granules with a small sachet 3 daily for 24 weeks, after breakfast, lunch and dinner respectively. The granules of each sachet will be dissolving in 100 ml boiled water.

Control group: patients in the control group will be given placebo granules with a small sachet 3 daily for 24 weeks. The proceeding will be similar with the experiment group.

### 3.2 Outcomes measure

The following outcomes for the subjects will be assessed by independent researchers who do not know the allocation.

Primary outcomes: The primary outcomes are the change from the baseline in FVC at week 4, 12, 24 and times of acute exacerbations at week 4, 12, 24.

Secondary outcomes:

1. The change from baseline in the St. George's respiratory questionnaire total score at week 4, 12, and 24.
2. The change from baseline in forced expiratory volume in 1 second percentage/FVC at week 4, 12, and 24.
3. The change from baseline in diffusing capacity of Carbon monoxide at week 4, 12, and 24.
4. The change from baseline in brain natriuretic peptide at week 4, 12, and 24.
5. The change from baseline in curative effect of TCM syndrome at week 4, 12, and 24.

### 3.3 Safety assessment

With recorded in Treatise on Febrile Diseases, 1 of the 4 Classics of TCM, the Maimendong decoction has been used for nearly 2000 years. Chinese herbs dosage of the MMD in this clinical trial will be accordance with the People's Republic of China Pharmacopeia (2015 edition). Moreover, the vital signs and electrocardiogram of subjects will be monitored by doctors, and we will do laboratory tests including blood, urine, stool routine, liver function, renal function to assess the safety of MMD from the enrollment to the end of this study. Adverse events will be recorded at any time they occur during the treatment period.

### 3.4 Compliance of subjects

Researchers are responsible for contacting all the subjects during the study after enrollment. All patients will be required to return all empty containers of drugs at each follow-up visit. The costs of examination and transportation will be free. Researchers will explain the results of examinations to participants every visit. Patients will get the message from Wechat or phone before visit for the upcoming data collection. Moreover, treatment advises will be provided to the patients in the course of the study.

### 3.5 Adverse events

Adverse events of patients will be recorded in case report files. If a serious adverse event occurs, the intervention will be suspended immediately after evaluation of researchers. Then, we will keep records of the details about the time of occurrence, the severity of condition and the relation of drugs. All measures will be recorded in accordance with standard operational procedures of the China Food and Drug Administration. Besides, serious adverse events will be made known to the Steering Committee and Ethics Committee within a day.

### 3.6 Statistical analysis

The data will be analyzed by the SPSS V.23.0 (Chicago, IL) software package after the collection of data from the entire sample. All the *P*-values will be for 2-sided distributions, with a significant level of .05 for α. A descriptive analysis of the baseline date of the sample will be performed. All the quantitative variables will be analyzed by the Kolmogorov-Smirnov test with Lilliefors corrections, to ascertain whether the variables follow a normal distribution or not. The initial homogeneity between the groups will be also analyzed (ANOVA or Kruskal–Wallis depending of the normality of data distribution).

The measurement data will be examined using group *t*-tests or non-parametric tests, the count data will be tested using a Chi-square test or Fisher exact probability method, and the grade data will be tested using nonparametric tests.

All data analyses will be conducted with the intention to treat principles. If there are losses of follow-up, the outcome variables that have not been recorded will be completed with the last data recorded for each subject of these data.

## 4 Discussion

There are 2 different theories in TCM and Western medicine about recognition and treatment of diseases. Diagnosis of TCM will combine disease, syndrome, relevant clinical signs and symptoms, with all together to establish a treatment principle. Syndrome differentiation is the core of TCM practice, and we formulate the MMD to treat IPF patients for this study with qi and yin deficiency syndrome.

IPF is a serious global health problem with poor prognosis. Available medications for IPF cannot get the satisfactory results for patients at present. There have been many years of TCM prescription used in adjuvant treatment for IPF in China. MMD is a common formula for the disease.

As we know, this is the first double-blind, placebo-controlled, randomized clinical trial to evaluate the efficacy and safety of MMD plus western medicine in the treatment of IPF. This study will provide a preliminary evaluation of the efficacy and safety of MMD in IPF. Sixty subjects will be recruited in this clinical study. At the same time, they will be divided into 2 groups according to the randomization performed before recruitment. However, there are some limitations in the study. On the one hand, the sample size is too small, which calculated based on previous research, and it may be smaller than it needs. On the other hand, a follow-up period of 24 weeks may be relatively short duration to assess the efficacy and safety of TCM in IPF, a chronic disease.

In conclusion, this study hypothesis that MMD plus Pirfenidone can improve pulmonary function, decrease the time of acute exacerbations and improve quality of life with IPF patients. If the results of the study are positive, it will be possible to give another option in the area of adjuvant treatment with IPF.

## 5 Others

### 5.1 Trial status

The study will begin from September, 2020 and end in September, 2022. In this trial, a total of 60 participants will be recruited.

## Acknowledgments

Thanks to Dr. Chuantao Zhang for his assistance and valuable advice. We are also grateful to “Xinglin scholar” Research Premotion Project of Chengdu University of Traditional Chinese Medicine (XSGG2019016), “100 Talent Plan” Project of Hospital of Chengdu University of Traditional Chinese Medicine (Hospital office [2020] 42) and National training program for innovative backbone talents of traditional Chinese Medicine (No. 91 [2019] of the State Office of Traditional Chinese Medicine) for their support.

## Author contributions

**Conceptualization:** Tingting Liao.

**Investigation:** Qun Huang, Niao Huang.

**Supervision:** Jundong Wang, Chuantao Zhang.

**Writing – original draft:** Wenfan Gan, Yuancheng Liang.

**Writing – review & editing:** Guojin Xiao, Ying Luo.
